# Hémophilie B mineure révélée par une hémorragie cérébrale: à propos d'un cas

**DOI:** 10.11604/pamj.2015.21.76.5875

**Published:** 2015-05-29

**Authors:** Abdelhalim Naji, Maria Rkain, Rim Amrani, Noufissa Benajiba

**Affiliations:** 1 Service de pédiatrie, CHU Mohammed VI Université Mohammed Premier Oujda, Maroc

**Keywords:** convulsion néonatale, pâleur, hémorragie cérébrale, neonatal convulsion, paleness, cerebral hemorrhage

## Abstract

L'hémorragie intracrânienne (HIC) du nouveau-né à terme est une pathologie rare, leur prévalence est estimée à 2% des naissances vivantes. Les manifestations cliniques sont variables et non spécifique. Les causes d'HIC sont multiples et souvent intriquées, les mécanismes physiopathologiques principaux sont la dysrégulation du débit cérébral, une obstruction des vaisseaux ou une coagulation intravasculaire; ou une lésion directe par traumatisme. Nous rapportons le cas d'un nourrisson d'un mois qui a été admis dans notre service pour prendre en charge des convulsions associées à une pâleur cutanéomuqueuse, suite à laquelle l'examen biologique a mis fortuitement en faveur une hémophilie mineure sur une maladie hémorragique tardive.

## Introduction

Les hémorragies constituent une urgence néonatale fréquente, pouvant résulter de plusieurs causes, soit d'un trouble de l'hémostase primaire; ou d'un trouble de la coagulation. La maladie hémorragique du nouveau-né constitue la cause la plus fréquente [1]. Nous rapportons l'observation d'un nourrisson d'un mois chez qui la survenue d'un hématome cérébral suite à une maladie hémorragique tardive a révélé une hémophilie B mineure.

## Patient et observation

**Figure 1:**
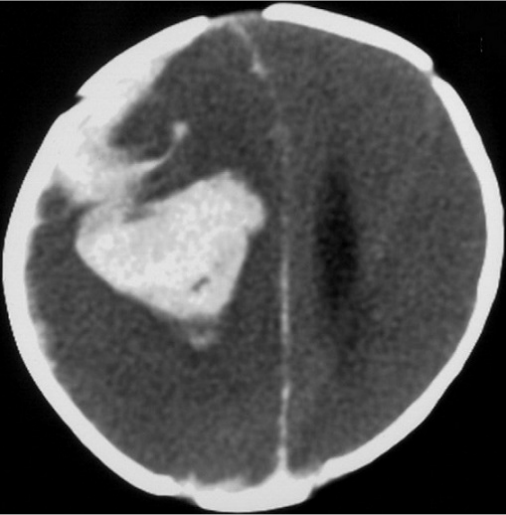
Scanner cérébral démontrant le nouveau saignement, une lame d'hématome sous-dural aigu avec hématome intra parenchymateux fronto-pariétal droit

Il s'agit d'un nourrisson âgé d'un mois issu d'un mariage consanguin sans antécédents familiaux particuliers, notamment pas d'hémophilie, la grossesse était mené à terme sans incidents avec un accouchement médicalisé non instrumental, sous allaitement maternel exclusif, le nourrisson n'a pas reçu sa dose prophylactique de vitamine K. Il a été admis pour convulsion tonico-clonique localisée à l'hémicorps gauche, l'examen avait noté une fièvre à 38, une pâleur cutanéomuqueuse et des ecchymoses au niveau du dos. L'examen neurologique a retrouvé une asymétrie de tonus, une macrocranie avec un périmètre crânien à 43 cm, et des yeux en coucher de soleil. le diagnostic d'hémorragie cérébrale sur un trouble d'hémostase a été suspecté. Une TDM cérébrale a été réalisée révélant un hématome cérébral et une hémorragie méningée (Figure 1). Le bilan biologique a révélé une anémie avec un taux d'hémoglobine à 10 g/100ml, et un taux de plaquettes normal. Le bilan d'hémostase était perturbé avec un TP bas et un TCA allongé Le nourrisson a été mis sous vitamine K injectable à raison de 10 mg et transfusé en Plasma frais congelé, mis sous anticonvulsivants, et antibiotiques à base de C3G dose méningée. Durant son hospitalisation, l’évolution a été marquée par la normalisation de l'hémostase quelques heures après administration de la vitamine K, On note aussi la disparition des ecchymoses, avec stabilisation de l’état neurologique, le périmètre crânien est passé à 44cm à J8, la TDM cérébrale de contrôle a noté une résorption de l'hématome et une hydrocéphalie tri ventriculaire modérée (Figure 1) qui n'a nécessité qu'une surveillance neurochirurgicale régulière. Un bilan d'hémostase avec dosage des facteurs de coagulation fait à un mois d'intervalle de la transfusion a révélé un taux de facteur IX à 36% (VN 50 à 150) et tous les autres facteurs présentaient des taux normaux), ce qui était en faveur d'une hémophilie B mineure, qui n'expliquait pas la sévérité du tableau clinique.. L’évolution a été favorable. Le nourrisson a été revu en consultation avec un recul de 4 mois et son examen neurologique était normal pour l´âge. Il s'agissait donc d'une hémophilie B mineure révélée à la période néonatale par un hématome cérébral sans notion de traumatisme, du probablement à la maladie hémorragique tardive.

## Discussion

Les troubles de la coagulation représentent 32.1% des étiologies des hémorragies cérébrales, après les troubles vasculaires (et infarctus hémorragique, thromboses veineuses, malformations vasculaires) et les accidents à la naissance (encéphalopathies anoxo-ischémiques, traumatismes obstétricaux), et sont de plusieurs types donnant des tableaux cliniques et des lésions différentes [2]. La maladie hémorragique tardive est essentiellement caractérisée par la survenue d´une hémorragie cérébrale, au premier mois de vie où l´on observe une absence de prophylaxie néonatale par vitamine K, associée à une absence de supplémentation par vitamine K lors de l´allaitement maternel. En outre l'hémophilie, est l'affections la plus fréquemment à l´origine des hémorragies cérébrales. Elle entraîne la plupart du temps des hématomes sous-duraux mais aussi des hémorragies parenchymateuses ou cérébelleuses se compliquant souvent de séquelles. Ces hémorragies cérébrales surviennent chez 1 à 4% des naissances des enfants hémophiles [3, 4]. La fréquence des hémorragies intracrâniennes (HIC) néonatales occasionnées par l'hémophilie a été longtemps sous-estimée sans doute du fait de la mauvaise prise en compte des nombres de décès et de l'absence de diagnostic d'hémophilie posé dans ces circonstances [5]. Le risque hémorragique (HIC et/ou cephal hématome) est majoré par les manœuvres instrumentales d'extraction (forceps, ventouse). Les signes cliniques sont précoces survenant dans les premiers jours de vie [3]. Il faut donc savoir dépister les facteurs de risques familiaux afin d´adapter le mode d´accouchement en cas de doute. Par ailleurs, l´hémorragie cérébrale pouvant être un mode de révélation d´une hémophilie, il faut donc savoir la rechercher devant toute HIC inexpliquée, voire même ne pas hésiter à la traiter comme telle en attendant la preuve diagnostique. la sévérité des manifestations hémorragiques est corrélée à celle du déficit en facteur de coagulation. Les manifestations hémorragiques des formes sévères sont spontanées ou surviennent à l´occasion d´un traumatisme minime [6, 7]. Dans les formes modérées et mineures, les saignements sont occasionnels et provoqués par un traumatisme ou un acte chirurgical. Dans notre cas, la sévérité du tableau clinique et l'absence d'utilisation de manœuvres instrumentales lors de l'accouchement ne peuvent pas être expliquées par le degré de déficit de facteur IX, ce qui laisse suggérer, vu aussi l'absence de prophylaxie par la vitamine K, que la maladie hémorragique néonatale dans sa forme tardive reste l’étiologie la plus probable de ce saignement, associé chez ce malade à une hémophilie B mineure.

## Conclusion

La maladie hémorragique du nourrisson reste un problème en pédiatrie. La vitamine K administrée abaisse significativement la prévalence de cette pathologie, y compris chez les enfants exclusivement allaités chez qui lerisque est plus élevé.
